# First Characterisation of the *Phoma* Species Complex on Maize Leaves in Central Europe

**DOI:** 10.3390/pathogens10091216

**Published:** 2021-09-18

**Authors:** Lucia Ramos Romero, Dagmar Tacke, Birger Koopmann, Andreas von Tiedemann

**Affiliations:** Section of Plant Pathology and Crop Protection, Department of Crop Sciences, Faculty of Agricultural Sciences, Georg August University, Grisebachstraße 6, 37077 Göttingen, Germany; Lucia.Ramos@plantandfood.co.nz (L.R.R.); dtacke@gwdg.de (D.T.); bkoopma@gwdg.de (B.K.)

**Keywords:** *Phoma* species complex, *Didymella* spp*. Zea mays*, maize leaf spots, phylogenetic analysis, European maize production

## Abstract

In the last decade, the cultivated area of maize has increased in Central Europe due to its high yield potential and diverse uses for feed and bio-energy. This has led to more intense maize cultivation, with narrowed crop rotations resulting in the increase in maize leaf diseases. During 2012 and 2013, an inventory of maize leaf spot diseases was carried out in various regions in Central Europe. In addition to the major leaf pathogens, isolates of *Phoma*-like species were obtained from oval to elliptical spots on leaves or found in lesions produced by other leaf pathogens. A total of 16 representative *Phoma*-like strains were characterised for their pathogenicity on maize leaves, for their morphological characteristics and with a phylogenetic analysis based on multilocus sequence analysis using part of the actin (ACT), calmodulin (CAL), β-tubulin (TUB), internal transcribed spacer (ITS) region of ribosomal DNA and large subunit ribosomal RNA (LSU) genes. The strains were grouped into four clades, and morphological studies supported this classification for most of them. Strains were compared with six reference *Phoma*-like species strains from the Westerndijk Fungal Biodiversity Institute collection reported to colonise maize. The pathogenic group of strains from our collection (after completion of Koch’s postulates) did not cluster with any of these species, indicating a different and novel *Phoma*-like species infecting maize leaves. To our knowledge, this is the first study dissecting the *Phoma* species complex on maize leaves in Central Europe.

## 1. Introduction

In the last decade, the cultivated area of maize has progressively increased in Central Europe due to its high yield potential and the option of diverse uses for feed and bioenergy. This has led to more intense maize cultivation in many regions, with narrowed crop rotations resulting in the increased presence of various maize leaf diseases such as northern corn leaf blight (*Exserohilum turcicum*, teleomorph *Setosphaeria turcica*) and eyespot (*Kabatiella zeae*). In order to monitor the current situation, an inventory of maize leaf spot diseases was carried out in 2012 and 2013 in various maize cultivating regions of Central Europe. In addition to the major leaf pathogens, 21 different *Phoma*-like samples were obtained from oval to elliptical spots on leaves or were found in lesions produced by other maize leaf pathogens.

The genus *Phoma* and other related genera are known to contain a large number of pathogenic species associated with leaf, grain or stem diseases. These have been shown to damage many important crops worldwide such as citrus lemon [1], sunflower [2,3], oilseed rape [4,5,6], tobacco [7], sorghum [8,9], wheat [10,11], rice [12], sugarcane [13] and coffee [14], potentially causing significant economic losses. Some *Phoma* species or fungi related to this genus have also been reported as causal agents of diseases on leaves, stems, roots and grains of maize [15,16,17,18,19,20,21].

*Phoma* and *Phoma*-related diseases on maize leaves, such as yellow leaf blight caused by *Didymella maydis* Arny & R.R. Nelson (syn. *Phoma zeae-maydis* Mukunya & Boothr), have been widely reported in the last century in temperate regions of the USA [15,22,23,24,25] and Canada [26]. More recently, further *Phoma*-like species causing foliar diseases on maize leaves were reported more frequently from warm or tropical regions, including *Phaeosphaeria maydis* and *Epiccocum ovisporum* (described as *E. sorghinum;* syn. *Phoma sorghina*) as potential disease complexes, in which other *Phoma*-like species could also be involved [16,21,27,28].

To date, there is limited knowledge about the range of *Phoma* species occurring on European maize. Furthermore, it is still unclear which species are pathogenic on maize leaves, and whether they may cause biomass losses through a reduction in the photosynthetically active leaf area. The fact that *Phoma* spp. occurs as a species complex on maize leaves makes it difficult to comprehend the role and importance of individual *Phoma* spp. as causal agents of leaf diseases. A proper identification therefore is, imperative, for evaluating pathogenicity and assigning disease symptoms to a causal agent.

Due to the large number of species comprised in the form complex *Phoma* (more than 3000), morphological and molecular identification and adequate classification of *Phoma* spp. is difficult [29,30,31]. Members of the *Phoma* complex are usually classified based on their respective host plant and detailed morphological characteristics. However, morphological characteristics may vary greatly with regard to in vitro culture conditions [30]. Therefore, additional molecular analysis is necessary for reliable identification. Regarding *Phoma* spp. derived from maize, several species have been reclassified recently in the genus *Didymella* [32,33].

The objective of the present work was to analyse the complex of *Phoma* spp. and *Phoma*-related genera collected from maize leaves based on cultural, morphological and molecular characteristics, as well as to assess their pathogenicity on maize. To our knowledge, this is the first study analysing in detail the *Phoma* complex present on maize in Central Europe.

## 2. Results

### 2.1. Collecting Fungal Isolates from Symptomatic Leaves

**Figure 1:**
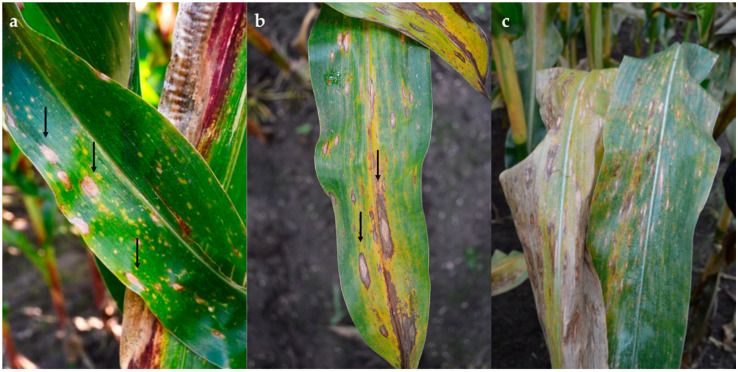
Symptoms of *Phoma*-like disease on naturally infected maize leaves. (**a**,**b**) Typical round lesions, lesions coalescing in large oval areas and leaf turning yellow; (**c**) leaf decay.

In the field, early symptoms on infected leaves associated with *Phoma*-like pycnidia and conidia consisted of round to oval lesions with an orange centre and yellow halo. These later expanded along the leaf veins, coalescing in large oval areas with a brown margin and grey centre, and a dark ring in the centre of the lesion (Figure 1a–c).

After incubation of the lesions in a moist chamber, numerous *Phoma*-like pycnidia emerged from the lesions (Table 1). Morphological differences among some isolates were distinguishable through light microscopy. Several different pycnidial *Phoma*-like isolates were sharing symptomatic leaves or were found in lesions produced by other maize leaf pathogens, which is indicated in Table 1. On symptomatic leaves from Ostenfeld, in addition to pycnidia, pseudothecia were observed and isolated on agar. However, further mycelial growth in vitro only produced pycnidia (strain 13.2P). A total of 16 representative *Phoma*-like isolates were selected for phylogenetic analysis based on multilocus sequence analysis (MLSA) using part of the actin (ACT), calmodulin (CAL), β-tubulin (TUB), internal transcribed spacer (ITS) region of ribosomal DNA and large subunit ribosomal RNA (LSU) genes.

*Phoma*-like symptoms were not observed in any of the six fields in the Czech Republic, and in the samples infected with other leaf pathogens (e.g., *B. zeicola* and *Puccinia* spp.), no *Phoma*-like pycnidia or spores were recovered. Only symptomatic samples infected with *E. turcicum* and *Colletotrichum graminicola* were received from France and Poland, and none of these presented *Phoma*-like pycnidia.

### 2.2. Morphology and Cultural Characteristics Related to Phylogenetic Clades

**Figure 2:**
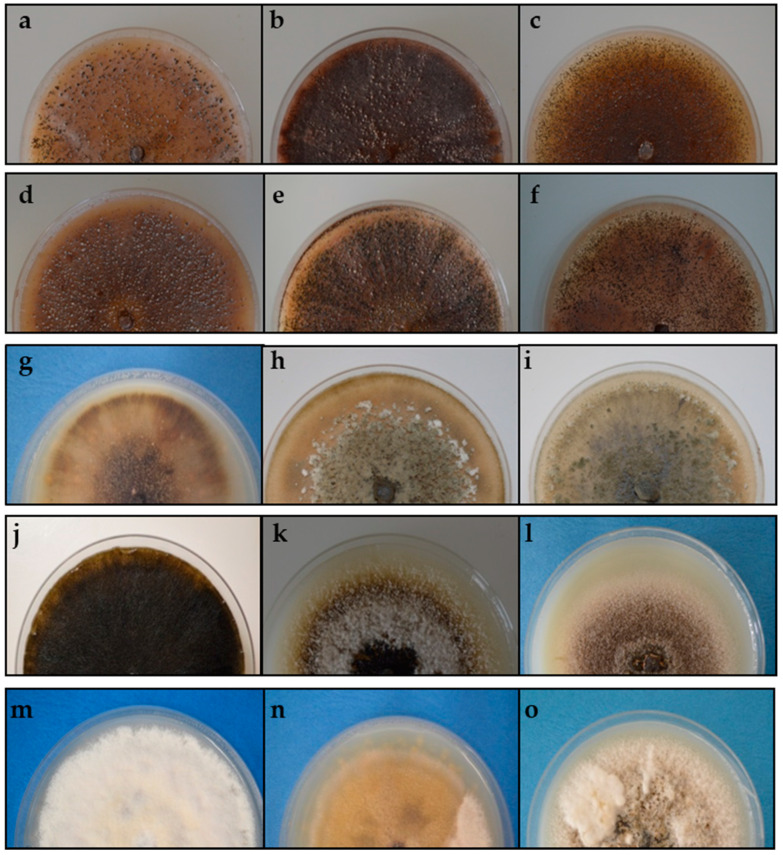
Colonies of some representative strains of *Phoma*-like species clades on oatmeal agar after incubation for 14 days in complete darkness at room temperature (18 to 20 °C). (**a**–**f**) Clade A: strains 13.2d (**a**), 12.13 (**b**), 12.27 (**c**), 12.28 (**d**), 12.31 (**e**), 13.47 (**f**); (**g**) Clade B: 13.2B; (**h**–**j**) Clade C: strains 13.36 (**h**), 13.37 (**i**), 12.10 (**j**); (**k**–**o**) Clade D: strains 12.18 (**k**); 12.20 (**l**), 13.2P (**m**), 13.2C (**n**), 12.23.1p (**o**).

**Figure 3:**
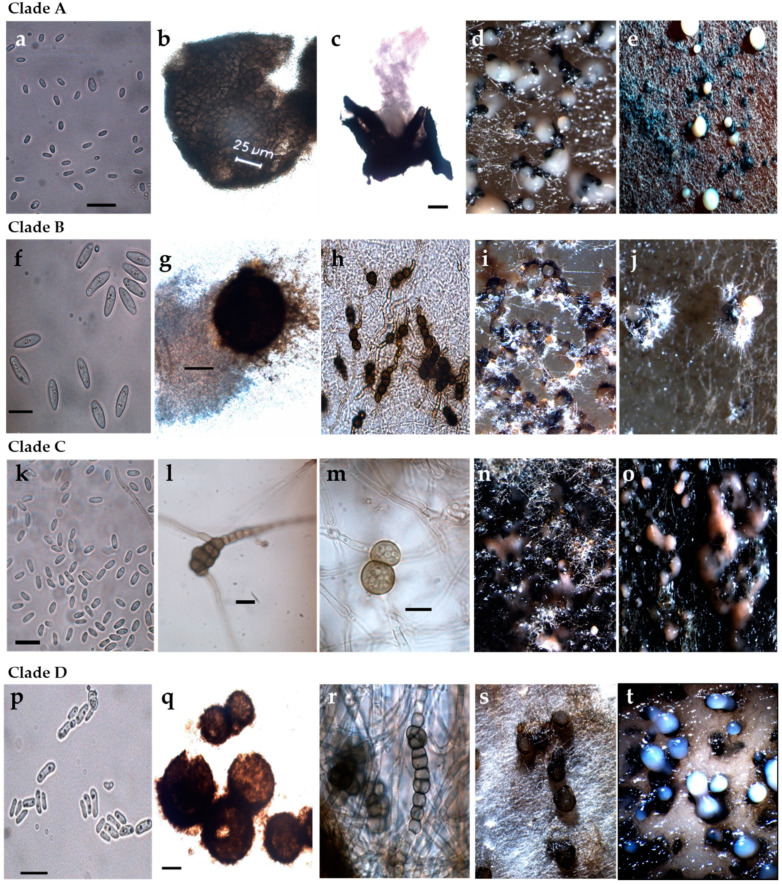
Macro- and micromorphological features of selected representative strains for each *Phoma*-like clade. (**a**–**e**) Clade A (strain 12.13 and 12.27); (**f**–**j**) Clade B (strain 13.2B); (**k**–**o**) Clade C (strain 13.36 and 13.37); (**p**–**t**) Clade D (strains 12.18, 12.20 and 13.2P); (**a**,**f**,**k**,**p**) Conidia; (**b**,**c**,**g**,**q**) Pycnidia; (**h**,**l**,**m**,**r**) Chlamydospores and dictyochlamydospores; (**d**,**e**,**i**,**j**,**n**,**o**,**s**,**t**) Pycnidia and conidiomata on oatmeal agar. Scale bars conidia (**a**,**f**,**k**,**p**) = 10 µm. Scale bars pycnidia (**c**,**g**,**q**) = 100 µm. Scale bars chlamydospores (**l**,**m**) = 10 µm.

Strains that were identified based on the phylogenetic tree analysis using the concatenated data were also identified on the basis of colony morphology and conidial characteristics in oatmeal agar (OA). The cultural characteristics of strains on OA significantly varied between different clades (Table 2, Figure 2 and Figure 3).

The phylogenetic analysis grouped strains 13.2d, 12.13, 12.27, 12.28, 12.31, 13.47 and 13.48 in a common clade, which also had similar cultural and morphological characteristics. Colonies had regular margins with sparse production of aerial mycelium, salmon to hyaline, flat to effuse and scattered. Immersed mycelia were dark brown or red to vinaceous, making the colony clearly identifiable. Pycnidia were produced abundantly and homogeneously distributed on the plate. Unusually large spores were observed in some strains.

Strains 13.36 and 13.37 clustered together and also exhibited similar morphological features. Both strains had a colony growth rate varying between 54 and 58 mm after 7 days, with irregular to regular margins and a moderate to abundant production of green to olivaceous buff, floccose to woolly aerial mycelium with floccose white tufts. Dictyochlamydospores were usually present. Phylogenetic analysis also grouped strain 12.10 with these two strains, although no similarity was found in cultural and morphological characteristics. Colonies of strain 12.10 were dark with immersed mycelium covered with grey to brown, flat to effuse aerial mycelium and with a slower growth pattern (39 to 43 mm after 7 days). Black pycnidia were abundantly produced, both on the surface and in the agar. Immersed mycelium and pycnidia produced dark discolorations of the agar medium.

Strain 13.2B formed an own clade. Colonies were regular and grew 35 to 38 mm in diameter within 7 days and exhibited scarce production of brown, flat to effuse aerial mycelium, which developed into some white floccose areas in the centre. A key characteristic was the reduction in mycelium production under near-UV light, where mostly pycnidia were produced. Compared with all the strains of this study, the conidia were distinctively larger.

Similarly, a further group deriving from phylogenetic analysis, strains 12.18, 12.20, 13.2P, 13.2C and 12.23.1p clustering in clade D, displayed similar cultural and morphological characteristics. Chlamydospores and dictyochlamydospores were observed in strains 13.2P and 13.2C, respectively. Strains in this clade were characterised by a slow in vitro growth rate (32 to 34 mm in 7 d) and scarce to moderate production of whitish, green to olivaceous floccose mycelium. This group was categorised as pathogenic after completion of Koch’s postulates (Section 2.4). It is noticeable that conidia in this clade were distinctively smaller (4.1–7.9 μm × 1.5–3.2 μm) than those obtained for the most common pathogenic *Phoma*-like species, *D. maydis* (CBS 588.69) (10.8–16.5 μm × 3.7–4.8 µm).

### 2.3. Phylogenetic Analysis

Sixteen strains of *Phoma*-like species were selected as representatives for MLSA (Table 1). These strains were analysed together with the six reference *Phoma*-like species strains from the WFBI collection. These reference strains were chosen for classification as they are reported in the literature to colonise maize. 18S ribosomal RNA gene amplicons generated by the use of NS primers showed no polymorphisms among the studied strains, which were therefore excluded from further analyses. Phylogenetical analysis of the ACT, CAL, TUB, ITS and LSU genes were employed for typing of all strains. This analysis involved nucleotide sequences of 22 strains with a total of 3006 positions in the final datasets.

**Figure 4:**
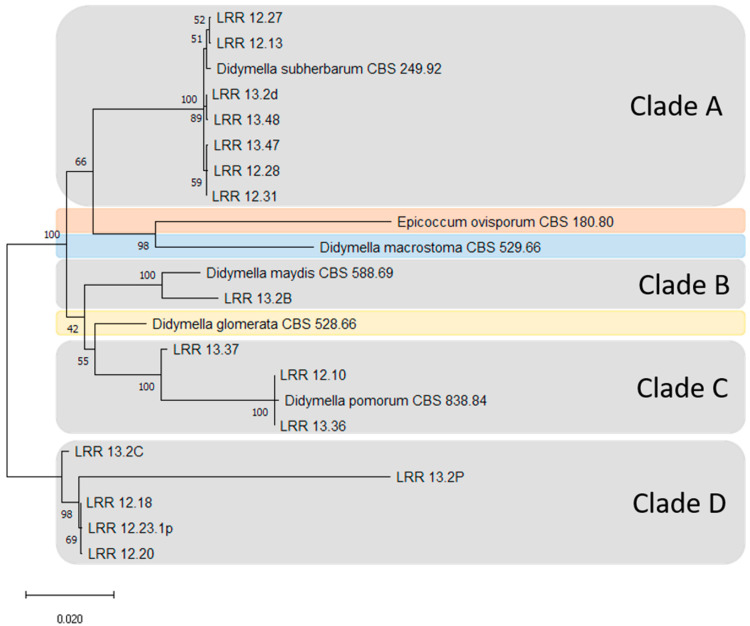
Dendrogram resulting from the concatenated analysis of part of the actin (ACT), calmodulin (CAL), β-tubulin (TUB), internal transcribed spacer (ITS) region of ribosomal DNA and large subunit ribosomal RNA (LSU) genes (using this order) sequence data following the maximum likelihood method and Tamura–Nei model (1993). The tree with the highest log likelihood (−9543.25) is shown. The percentage of trees in which the associated taxa clustered together is shown next to the branches. Initial tree(s) for the heuristic search were obtained automatically by applying Neighbour-Join and BioNJ algorithms to a matrix of pairwise distances estimated using the Tamura–Nei model, and then selecting the topology with superior log likelihood value. The scale bar represents the number of substitutions per site. This analysis involved 22 nucleotide sequences. There were a total of 3006 positions in the final dataset. Evolutionary analyses were conducted using the software Mega X [34]. The five strains in Clade D did not cluster with any of the known *Phoma*-like strains reported on maize from the Westerndijk Fungal Biodiversity Institute. This suggests a clade represented by a potential new species colonising maize.

The strains analysed in this study were grouped into four clades (Clades A–D) (Figure 4). All strains that were grouped based on colony morphology and conidial characteristics on OA corresponded with the clade clustering as well. The field strains were grouped as follows: seven strains of *Didymella subherbarum* (CBS 249.92) in Clade A, one with *D. maydis* (CBS 588.69) in Clade B and three with *Didymella pomorum* (CBS 838.84) in Clade C. The five strains in Clade D did not cluster with any known *Phoma*-like species recorded on maize leaves obtained from the WFBI. Strains belonging to Clades A and B and most of the strains from Clade D (with the exception of strain 12.18) were obtained from fields in northern Germany, while strains belonging to Clade C were obtained from southern Germany or Austria.

### 2.4. Pathogenicity Tests

**Figure 5:**
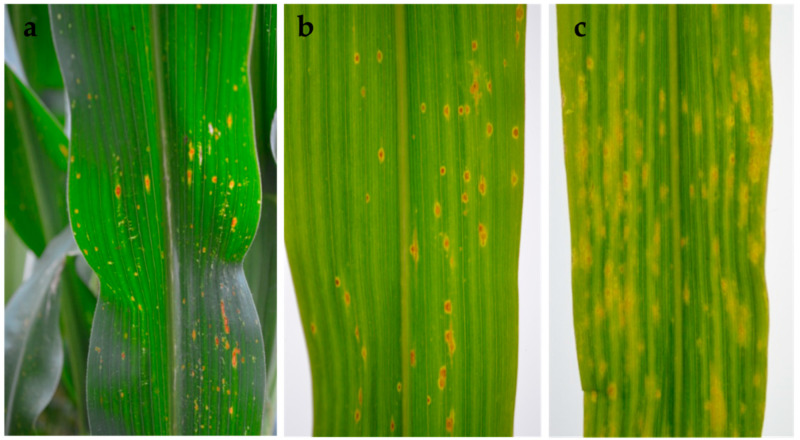
Symptoms of *Phoma*-like diseases on maize leaves. (**a**) Typical early lesions in the field and (**b**) after artificial inoculation of healthy plants in the greenhouse; (**c**) spots and large chlorotic areas produced by *Didymella maydis* (CBS 588.69), the causal agent of yellow leaf blight, obtained from the Westerndijk Fungal Biodiversity Institute. Inoculations were performed using maize seedlings from two varieties, Ricardinio and Barros (KWS SAAT SE, Einbeck, Germany), at leaf stage five to six.

Pathogenicity of the different *Phoma*-like strains was assessed on leaves of two maize varieties (Ricardinio and Barros) with conidial suspensions. For five strains (12.18, 12.20, 13.2P, 13.2C and 12.23.1), chlorotic lesions (1 to 2 mm) were observed on both maize varieties 2 to 3 days after inoculations. Thereafter, the chlorosis developed into round to oval lesions with an orange centre that turned necrotic. Lesions were surrounded by a yellow halo expanding along the leaf veins, resembling the initial symptoms in the field (Figure 5). Furthermore, strains 12.18 and 12.20 induced premature death of the leaves. Among the strains obtained from the Westerndijk Fungal Biodiversity Institute (WFBI), only *D. maydis* (CBS 588.69) produced lesions. Although these lesions were similar to those obtained with our pathogenic strains from the field, they exhibited a larger chlorotic area. All these strains (12.18, 12.20, 13.2P, 13.2C, 12.23.1p) and *D. maydis* (CBS 588.69) were recovered from lesions in the infected tissue, resembling the initially inoculated conidia, hence fulfilling Koch’s postulates and being considered pathogenic. Noninoculated control plants remained asymptomatic.

The following strains were considered nonpathogenic: strain 12.3B, which developed chlorosis on leaves of both maize varieties, but these did not turn necrotic; strains 13.36 and 13.37, which produced very slight chlorosis to the upper leaf on the Ricardinio maize variety only; and the rest of the strains obtained during the assessment (12.10, 13.2d, 12.13, 12.27, 12.28, 12.31, 13.47, 13.48), which were unable to produce any chlorotic or necrotic lesions.

The five pathogenic strains obtained from maize fields (Clade D) did not cluster with any of the nonpathogenic strains (Clades A and C). Furthermore, the pathogenic strains did not cluster with any of the known *Phoma*-like species recorded on maize from the WFBI collection (pathogenic and nonpathogenic). This indicates that Clade D represents a novel *Phoma*-like species infecting maize that is different from any other strain analysed during this study. The field strains which were considered nonpathogenic were grouped as follows: seven with *D. subherbarum* (CBS 249.92) in Clade A, three with *D. pomorum* (CBS 838.84) in Clade C and one with *D. maydis* (CBS 588.69) in Clade B.

## 3. Discussion

Due to limited knowledge of the range of *Phoma* and *Phoma*-related species occurring on maize leaves in Central Europe, a consensus on which species are pathogenic and have the potential to cause economic losses is lacking. The pathogenic strains obtained in this work induced leaf spots in the greenhouse after spray inoculation, which were similar to the early symptoms found in the field and are described in the literature for the most common *Phoma*-like pathogen on maize leaves, *D. maydis* (first described as *Phyllosticta maydis*) [15]. In contrast, our morphological analysis showed that the conidia of our pathogenic strains were significantly smaller (4.1 to 7.9 μm × 1.5 to 3.2 μm) than those of *D. maydis* (CBS 588.69) (10.8 to 16.5 μm × 3.7 to 4.8 µm).

In addition to the morphological studies, a phylogenetic analysis using sequence data from the five loci ACT-CAL-TUB-ITS-LSU confirmed that our pathogenic representative strains differ from *D. maydis*. The multi-locus phylogeny provided a species-level recognition of *Phoma*-like species and confirmed that our pathogenic strains represent a group to be separated from those *Phoma*-like species provided by the WFBI collection and reported in the literature to colonise maize.

After artificial inoculation on maize seedlings, the pathogenic *Phoma*-like strains produced leaf lesions, which resembled the early lesions associated with *Phoma*-like species in the field. Further development of the spots into larger lesions, also similar to those observed in the field, could not be recorded. It is possible that the testing conditions differed from the real environmental conditions, and were suboptimal for the expansion of the lesion. Another possibility is that the varieties used for artificial inoculations in this work showed some degree of tolerance. To reproduce the typical lesions in an advanced stage, further studies could test different environmental conditions for infection (e.g., climatic conditions and plant stage), as well as other maize varieties.

Although chlorosis on leaves was only observed after inoculation with strain 13.2B, our phylogenetic analysis grouped this strain into the same clade with the leaf pathogen *D. maydis* (CBS 588.69). These results were supported by morphological analysis, which showed that conidia of strain 13.2B were particularly large, resembling in size and shape the measurements to the maize pathogen *D. maydis* (CBS 588.69). The spore density used with strain 13.2B (at 10^6^ conidia/mL levels) was higher than for pathogenicity tests with *D. maydis* performed by other authors [35]. Thus, this should have been sufficient for the strain to induce symptoms. In addition, during parallel inoculations with strains obtained from WFBI (data not shown), *D. maydis* (CBS 588.69) was able to produce lesions at lower inoculum levels (at 10^4^ conidia/mL levels) than used for strain 13.2B. As is the case with the development of larger lesions stated above, the absence of further development of the chlorosis into spots with strain 13.2B could have been a result of tolerance to our field strain of the maize varieties used during this work, or suboptimal environmental conditions during the pathogenicity tests specific to this strain. Further tests with this strain on other maize varieties could shed more light on its pathogenicity and resolve potential uncertainties about its phylogenetic relation to *D. maydis*.

During our inventory, pseudothecia were found in one location. When these were isolated on agar, further mycelial growth only produced pycnidia. As an example, the pseudothecial stage of *D. maydis* has been reported from maize leaves in fields of North America [36] and to be homothallic [37]. This illustrates that other *Phoma*-like species occurring on maize leaves can produce the teleomorph stage, shedding more light on their life cycle in the field. Further sequence analysis of the mating type locus of the *Phoma*-like strains obtained during this work could also provide further insight into their evolution and life cycle.

Pathogenic *Phoma*-like species with smaller conidia occurring on maize, similar to some of the strains obtained in this work, have been scarcely reported in the literature [25,38,39,40,41]. Although very uncommon, one of these is *Phoma zeae* G.L. Stout (4 to 7 μm × 2–2.65 µm) [15,38,40]. To our knowledge, no detailed reports with morphological and phylogenetic descriptions are available for this species, neither a reference strain of this species nor DNA-sequence information. Thus, a confirmation whether our pathogenic *Phoma*-like strains correspond to this earlier described species will be difficult.

Additional pathogenic *Phoma*-related species reported on maize such as the potential disease complex *Phaeosphaeria maydis* and *Epiccocum ovisporum* were disregarded. These differ from the descriptions of symptoms and morphological characteristics given in this work and are mostly reported from warm or tropical regions [16,21,27]. Furthermore, the phylogenetic studies presented in this work and further morphological observations (not presented) separated the reference strain *E. ovisporum* (strain CBS 180.80) from all our pathogenic and nonpathogenic strains.

During our pathogenicity tests, all nonpathogenic strains were tested at the same elevated inoculum level (10^7^ conidia/mL), which was similar or above the level used with the pathogenic strains (10^6^ to 10^7^ conidia/mL). Furthermore, reference strains *D. pomorum* and *D. subherbarum* obtained from WFBI were grouped with our nonpathogenic strains and did not induce any lesions either (data not shown), supporting the saprophytic role of the two groups represented by clades A and C.

During this work, most of the pathogenic strains were isolated from typical spots on maize leaves attributed to *Phoma*-like species (with the exception of strain 12.18), and Koch’s postulates were confirmed. However, two strains which were obtained from such lesions, 13.47 and 13.48, were classified as nonpathogenic and grouped according to phylogenetic analysis within the nonpathogenic Clade A. This provides evidence that not all *Phoma*-like species present in a lesion must be pathogenic, as both pathogenic and saprophytic can be sharing leaves.

Although *D. pomorum* and *D. subherbarum* have been reported to be isolated from maize in the literature, no pathogenicity has been observed in our study on leaves [42,43,44,45,46]. To our knowledge, *D. subherbarum* has only been reported from maize in North and South America [30,31], while *D. pomorum* has been reported from Denmark to be capable of producing isocumarins [46]. This highlights that, although the strains grouped into the same clade with the reference strain of *D. pomorum* were not capable of inducing primary leaf damage, they may be a threat through producing secondary toxic metabolites.

*Phoma* was isolated from fields in Austria, Germany and The Netherlands. Although fields in the Czech Republic were also visited, no *Phoma*-like symptoms were observed. Furthermore, no *Phoma*-like pycnidia or spores were obtained from leaves infected with other pathogens. Further research is needed to ascertain whether *Phoma* species are also present in these regions. This research could include the study of environmental conditions, and the effect that different cultural practices (e.g., soil management strategies and alternative maize varieties) may have on its appearance, comparing with countries where *Phoma* was found.

Symptomatic samples from France and Poland were received from our collaborators. The fact that no *Phoma*-like pycnidia were found in these samples does not rule out the possibility that *Phoma* is present in these regions. Only samples from two locations in each region were received, showing *E. turcicum* and *C. graminicola* symptoms. Further research, involving visiting and monitoring fields in these regions, is necessary to determine whether *Phoma* is present.

This study has shown the high diversity of *Phoma* and *Phoma*-related species occurring on maize leaves in Central Europe, comprising both pathogenic (*Phoma* sp. and *D. maydis*) and nonpathogenic (*D. pomorum* and *D. subherbarum*) species. Furthermore, this study presents the first investigation on the morphology, phylogeny and pathogenicity of the *Phoma* species complex on maize in the studied regions. It should also be emphasised that, besides proper morphological description, the fulfilment of Koch’s postulates is of utmost importance in order to separate pathogenic from saprophytic species. This clarification is crucial to assess the role of *Phoma* spp. in European maize production, as well as to compare the situation with other maize-growing regions worldwide.

## 4. Materials and Methods

### 4.1. Isolation from Leaves and Culturing of Phoma-like Species

Maize leaves were collected in September and October 2012 and 2013 from selected fields in Germany (27 locations), the Netherlands (six locations), Czech Republic (six locations), Austria (three locations), France (two locations) and Poland (two locations) in order to perform a qualitative assessment of potentially leaf-infecting pathogens in maize. Of these fields, a total of 9 in Germany, 2 in Austria, 6 in The Netherlands and 6 in the Czech Republic were visited. For the rest of the locations, samples were sent by collaborators to the Division of Plant Pathology and Plant Protection of the University of Göttingen for further analysis (refer to the Supplementary Table S1).

Following initial microscopical observation, pycnidia-forming isolates were obtained with two different methods. In the first, symptomatic leaf tissue (1 × 1 cm) was surface-disinfected in 2% sodium hypochlorite, rinsed twice for 45 s in sterile water, dried, placed on synthetic nutrient-poor agar (SNA) (0.75 g of KH_2_PO_4_, 0.75 g of KNO_3_, 0.375 g of MgSO_4_, 0.375 g of KCl, 0.15 g of glucose, 0.15 g of saccharose and 11.25 g of agar per litre) and incubated at room temperature. The second method was based on the Waring blender technique [15,47]. In this case, samples showing lesions were segmented into small pieces (approx. 1 cm in length), washed under tap water, dried with absorbent paper, transferred to sterile water in a beaker and blended for five minutes. Three successive dilutions were made in sterile water (1:10) and 100 µL of the suspension was plated on SNA in Petri dishes. The plate was left to settle for one hour in a slanted position to remove the excess liquid from the suspension. The fungus was isolated from the colonies developed on the agar after approximately one to two days.

Pycnidia emerging from the two methods mentioned above were transferred onto a new SNA plate and incubated at room temperature. To obtain monosporic conidial isolates, conidia were streaked on SNA. Monosporic colonies were identified with a stereomicroscope and aseptically transferred to oatmeal agar (OA; 15 g of oat flour, 11.25 g of agar per litre). Conidial suspensions were stored at –20 °C in sterile 25% glycerol. All strains were lyophilised and deposited for long-term preservation at the Division of Plant Pathology and Plant Protection of the University of Göttingen.

### 4.2. Morphological and Cultural Characterisation of Selected Phoma-Like Strains

Morphological characterisation of the strains was carried out after 14 days of incubation on OA plates in complete darkness at room temperature (18 to 20 °C). Colony diameter was measured after 7 days. To ensure the production of pycnidia under these conditions, a duplicate plate of each strain was incubated in a near-UV light regime of 12 h per day (λ = ~400 nm) to stimulate their formation.

Macro- or micromorphological and cultural assessments were mostly referred to data from [30,31]. The shape and size of pycnidia (average of 10 samples) and conidia (average of 30 samples) and the presence of chlamydospores and dictyochlamydospores were registered. Furthermore, six *Phoma*-like species from the WFBI (Utrecht, The Netherlands) culture collection were compared morphologically with the isolated specimens (Table 3). The selection of these CBS strains was based on literature reports indicating their potential to colonise maize (either as saprophyte or pathogen). The selected species were: *Didymella glomerata* (strain CBS 528.66), *Didymella pomorum* (CBS 838.84), *Epicoccum ovisporum* (strain CBS 180.80), *Didymella subherbarum* (strains CBS 249.92), *Didymella macrostoma* (CBS 529.66) and *D. maydis* (strain CBS 588.69). WFBI freeze-dried strains were obtained in lyophilised form and revived on OA plates.

### 4.3. Growth of Fungal Isolates for DNA Extraction

Among the total number of isolates generated from infected maize leaves, individual strains were selected for MLSA studies (Table 1). They represent the individual morphotypes which have been found. Those strains were analysed together with the reference strains from the WFBI collection (Table 3) believed to be useful for classification.

### 4.4. DNA Extraction

DNA extraction was carried out according to [49] with some modifications. A preceding lysis step was implemented using lyticase in a sorbitol/citrate buffer. About 100 mg of lyophilised mycelium was transferred into 2 mL E-cups. Grinding of mycelium was achieved with the help of a 5 mm tungsten bead in a Retsch mixer mill (MM400, Retsch GmbH, Haan, Germany) at 28 Hz for 1 min. This step was repeated three times whereby the cooling of samples on ice and inverting of the tubes was performed in between each run. After homogenisation, 500 µL of lyticase solution (200 U/mL of lyticase (Sigma-Aldrich, Saint Louis, MO., USA: 25 kU, Cat#L2524); 100 mM of K-citrate pH: 8.0; 50 mM of EDTA pH: 8.0; 1 M of Sorbitol; 5 mM of TCEP) was added. Samples were mixed twice for 30 s at 20 Hz using the Retsch mill. Lysis was achieved during 30 min of incubation at 37 °C on a horizontal shaker. Samples were supplemented with 125 µL of 5 M of NaCl and carefully mixed. They were then mixed with 1 mL of extraction buffer (100 mM of Tris pH 8.0, 20 mM of EDTA, 2% CTAB, 1.2 M of NaCl and 5 mM of TCEP). The mix was incubated for 1 h at 60 °C and then cooled down to room temperature, transferred to 5 mL E-cups and 1.5 mL of chloroform/isoamyl alcohol (24:1, *v*:*v*) was added. The samples were properly mixed and spun in a microcentrifuge at 3000× *g* for 15 min. The aqueous layer was transferred into new vials, and 2.2 mL of dilution buffer (100 mM of Tris pH 8.0, 20 mM of EDTA, 2% CTAB) was added. The samples were again carefully mixed followed by an incubation at 60 °C for 30 min and then spun at 3000× *g* for 15 min. The supernatant was discarded and the DNA-pellet resuspended in 220 μL of high-salt TE (10 mM of Tris (pH 8.0), 2 mM of EDTA, 1 M of NaCl) with RNase A (50 μg/mL). Samples were incubated for 30 min at 60 °C. The solution was transferred to 1.5 mL E-cups, and 11 µL of MagAttract suspension G solution (Qiagen GmbH, Hilden, Germany) was added followed by 220 µL of 100% ethanol. Samples were gently mixed for 5 min to allow binding of DNA. The beads were trapped at the vial edge with a magnet and the ethanol solution was poured off. Beads were washed three times using 440 µL of washing buffer (30% TE, 70% ethanol). The beads were subsequently dried for 10 min at room temperature. The beads were carefully resuspended in 220 µL of TE buffer (10 mM of Tris, 1 mM of EDTA, pH 8.0) and incubated for 5 min at 60 °C. Finally, the beads were separated from the suspension by trapping them at the edge of the E-cup, and the resuspended DNA was transferred to new vials by pipetting.

### 4.5. PCR Amplification of Multiple Loci

For MLSA analysis, parts of actin, calmodulin, tubulin and ribosomal genes were used. Primer sequences and references together with the individual annealing and elongation conditions are listed in Table 4. About 50 ng of fungal DNA were used in final reaction volumes of 20–50 µL. Concentrations of phosphorylated primers were 1 µM each. Concentrations of 0.01 U/µL of Phusion High-Fidelity DNA Polymerase were used in combination with the 1× HF buffer supplied by the manufacturer (New England Biolabs, Frankfurt, Germany) to minimise error rates during amplification. Nucleotide concentrations were 0.2 mM each. Suitable annealing temperatures were individually determined on a T Professional Basic Gradient thermal cycler (Biometra, Göttingen, Germany). Annealing temperatures and PCR profiles are shown in Table 4.

### 4.6. Cloning and Sequencing of PCR Amplicons

Amplicons generated with phosphorylated primers were gel-purified using the Fast Gene Gel/PCR extraction kit (Nippon Genetics, Düren, Germany). They were cloned into the *Sma*I blunted plasmid vector pUC19 and subsequentially dephosphorylated by a CIAP treatment. The amplicons were ligated to the linearised, gel-purified vector in a 3:1 molecular ratio, and then transformed into 50 µL of DH5α *E. coli* chemical competent cells (Life Technologies GmbH, Darmstadt, Germany) applying conventional protocols described in [55]. Colonies were grown overnight at 37 °C and recombinant clones were picked on LB media supplemented with 100 ppm of ampicillin using the β-X-Gal screen. Plasmids were prepared using alkaline lysis and checked for successful uptake of amplicons by subsequent restriction digest and electrophoretic analysis. Plasmids were sent for both forward and reverse strand sequencing (Microsynth Seqlab, Göttingen, Germany) using the vector-based primers M13 uni-21 (TGTAAAACGACGGCCAGT) and M13 rev-29 (CAGGAAACAGCTATGACC).

### 4.7. Sequence Analysis

Forward and reverse sequences were assembled and vector and primer sequences clipped using the SnapGene^®^ software (GSL Biotech, Chicago, IL, USA). Sequence polymorphisms among the individual target genes were first analysed by sequence alignment using Clustal Omega [56]. 18S ribosomal RNA gene amplicons generated by the use of NS primers showed no polymorphisms among the studied strains and were thereby excluded from further analyses. The five remaining loci were concatenated using the order ACT-CAL-TUB-ITS-LSU (supplementary file). Genetic relatednesses were visualised among analysed strains using the software Mega X [34] by creating a phylogenetic tree. The maximum likelihood method and Tamura–Nei model [48] were used for this purpose. The tree with the highest log likelihood was created and checked by bootstrapping (*n* = 1000). There were a total of 3006 nucleotide positions in the final dataset.

### 4.8. Pathogenicity Tests

Inoculations were performed using maize seedlings from two varieties, Ricardinio and Barros (KWS SAAT SE, Einbeck, Germany), at leaf stage five to six. Conidial suspensions from *Phoma* cultures grown on OA for 8–10 days were collected with sterile water containing 125 ppm of Silwet^®^ Gold to improve leaf adhesion (Spiess-Urania Chemicals, Hamburg, Germany). After filtration through a sterile cheese cloth, the density was adjusted to a final conidia density of 1 × 10^6^ to 10^7^ spores/mL depending on the capacity of each strain to sporulate during the first and second inoculation (Table 5). Inoculations were performed using a pump sprayer. Control seedlings were inoculated with water and 125 ppm of Silwet^®^ Gold. Inoculated plants were incubated in a moist chamber and sprayed with water for 3 days before returning them to the greenhouse. Pathogenicity was assessed 14 days after inoculation. All pathogenicity tests were carried out twice. After development of lesions, symptomatic leaf samples were collected, disinfected, incubated on SNA plates at room temperature and examined for sporulation after 2 days under a stereomicroscope. If the characteristic pycnidia and conidia were recovered from lesions in the infected tissue, Koch’s postulates were considered completed and the strain was classified as pathogenic.

## Figures and Tables

**Table 1:** List of selected *Phoma*-like strains obtained from maize deposited at the Division of Plant Pathology and Plant Protection of the University of Göttingen and used in this study for multilocus sequence analysis.

IPP# ^1^	Description	Strain Designation ^2^	Location	Region	Isolated From ^3^
1562	*Phoma* sp.	LRR 12.18	Schönering	Northern Austria	3
1563	*Phoma* sp.	LRR 13.2B	Ostenfeld	Northern Germany	2
1564	*Phoma* sp.	LRR 13.2C	Ostenfeld	Northern Germany	2
1566	*Phoma* sp.	LRR 12.27	Bad Oldesloe	Northern Germany	3
1567	*Phoma* sp.	LRR 12.13	Stapel	Northern Germany	3
1568	*Phoma* sp.	LRR 12.10	Braunau	Northern Austria	4
1569	*Phoma* sp.	LRR 13.2d	Ostenfeld	Northern Germany	1
1570	*Phoma* sp.	LRR 13.48	Kassel	Central Germany	2
1571	*Phoma* sp.	LRR 13.47	Kassel	Central Germany	2
1572	*Phoma* sp.	LRR 12.28	Groningen	The Netherlands	3
1573	*Phoma* sp.	LRR 13.36	Mittich	Southern Germany	5
1574	*Phoma* sp.	LRR 13.37	Hartkirchen	Southern Germany	5
1575	*Phoma* sp.	LRR 12.23.1p	Rade	Northern Germany	2
1576	*Phoma* sp.	LRR 13.2P	Ostenfeld	Northern Germany	6
1577	*Phoma* sp.	LRR 12.20	Nordholz	Northern Germany	2
1585	*Phoma* sp.	LRR 12.31	Giekau	Northern Germany	3

IPP#—Collection number Institute for Plant Pathology, University Göttingen; ^1^ Internal collection number of the Institute of Plant Pathology, ^2^ For the sake of brevity, strain designations are used in the following mostly without preceding letters, ^3^ 1 = maize debris; 2 = lesions attributed to *Phoma*-like species; 3 = leaves with symptoms of *Kabatiella zeae*; 4 = leaves with symptoms of *Exserohilum turcicum*; 5 = leaves with symptoms of *Bipolaris zeicola*; 6 = obtained from pseudothecia on lesions attributed to *Phoma*-like species. LRR = Designations after Lucia Ramos Romero who obtained the isolates.

**Table 2:** Common and differential features of selected strains analysed in vitro according to [31]. Selected strains are grouped according to phylogenetical analysis. Clade A: 13.2d, 12.13, 12.27, 12.28, 12.31, 13.47, 13.48; Clade B: 13.2B; Clade C: 13.36, 13.37, 12.10; Clade D: 12.18; 12.20, 12.23.1p, 13.2P, 13.2C. Representation: ++ determining characteristic. + characteristic for the majority of strains. ± only found in some strains.

Clade	A	B	C	D *
**Pathogenic on maize**	no	no	no	yes
**Colony type**				
White-grey-green			+	±
Red/vinaceous/brown	++			
Brown		+	+	±
**Growth**				
Fast (45–75 mm)	++		+	
Moderate-slow (24–43 mm)		+	+	++
**Pycnidia**				
Glabrous	+		+	+
Pilose		++		
Form	Globose/subglobose/irregular/flask-shaped	Globose/subglobose/irregular	Globose/subglobose	Globose/subglobose/irregular
Solitary	+	+	+	+
Confluent	+		+	±
Conspicuous ostiole/necks	++/+			
Size (l × w) (µm)	70–300 × 75–250	200–500 × 100–400	50–200 × 45–200 **	50–350 × 50–300
Matrix color	Rosy-buff/white	Hyaline-white	Hyaline/pinkish	Hyaline/white
**Chlamydospores**				
Unicellular	±	+	±	±
Multicellular		+	±	±
**Conidia**				
Common size	Small	Large	Small	Small
Size (l × w)	2.5–6.3 × 0.9–2.4	9.9–11.9 × 3.3–4.3	3.8–6.5 × 1.6–2.3	4.1–7.9 × 1.5–3.2
Size (average)	3.7 × 1.7	10.9 × 3.9	4.9 × 2.3	4.7 × 2.4
Aseptate	+	+	+	+
Extrusion in cirri	+			
Gutules	0–2	1–5	0–5	1–5
Polar	+	+	+	+
Apolar		+		±

* Strain 12.23.1p characteristics only recorded for pathogenicity, colony type and growth. ** Size of the pycnidia based on strains 12.10 and 13.36.

**Table 3:** List of reference *Phoma*-like strains from the Westerndijk Fungal Biodiversity Institute collection used for multilocus sequence analysis. These strains were chosen for classification as they have been reported in the literature to colonise maize (as pathogenic or saprophytic).

IPP#	Description	Strain Code	Originator	Reference
1402	*Didymella maydis*	CBS 588.69	WFBI *	[32]
1403	*Epicoccum ovisporum*	CBS 180.80	WFBI	[48]
1404	*D. macrostoma*	CBS 529.66	WFBI	[32]
1405	*D. pomorum*	CBS 838.84	WFBI	[32]
1406	*D. glomerata*	CBS 528.66	WFBI	[32]
1462	*D. subherbarum*	CBS 249.92	WFBI	[32]

IPP#—Collection number Institute for Plant Pathology, University Göttingen; * Westerndijk Fungal Biodiversity Institute.

**Table 4:** Primers and PCR profiles used for multilocus sequence analysis.

Primer Name	Primer Sequence	Reference	Amplicon Sizes (bp)	PCR Profile
**Partial 18S ribosomal RNA gene (SSU)**			1133	98 °C, 10 s; 60 °C, 30 s; 72 °C, 90 s; (35×)
**NS1-fw**	GTAGTCATATGCTTGTCTC	[50]		
**NS4-rev**	CTTCCGTCAATTCCTTTAAG	[50]		
**Partial 28S ribosomal RNA gene (LSU)**			1421–1422	98 °C, 10 s; 60 °C, 30 s; 72 °C, 90 s; (35×)
**LR0R-fw**	GTACCCGCTGAACTTAAGC	[51]		
**LR7-rev**	TACTACCACCAAGATCT	[52]		
**5.8S nrRNA gene with the two flanking internal transcribed spacers (ITS)**			558–565	98 °C, 10 s; 59 °C, 15 s; 72 °C, 15 s; (35×)
**ITS-5-fw**	GGAAGTAAAAGTCGTAACAAGG	[50]		
**ITS-4-rev**	TCCTCCGCTTATTGATATGC	[50]		
**Partial actin gene (ACT)**			263–275	98 °C, 10 s; 66 °C, 30 s, 72 °C 15 s; (30×)
**ACT-512F-fw**	ATGTGCAAGGCCGGTTTCGC	[53]		
**ACT-783R-rev**	TACGAGTCCTTCTGGCCCAT	[53]		
**Partial beta-tubulin gene (Tub2)**			376–382	98 °C, 10 s; 63 °C, 30 s; 72 °C, 15 s; (30×)
**TUB2Fd-fw**	GTBCACCTYCARACCGGYCARTG	[54]		
**TUB4Rd-rev**	CCRGAYTGRCCRAARACRAAGTTGTC	[54]		
**Partial calmodulin gene (CAL)**			531–544	98 °C, 10 s; 63 °C, 30 s; 72 °C, 15 s; (35×)
**CAL-228F-fw-BK**	GAGTTCAAGGAGGCATTCTCTC *GAGTTCAAGGAGGCCTTCTCCC*	This study, modified from [53], original sequence in italics		
**CAL-737R rev-BK**	CATCTTGCGGGCCATCATAG *CATCTTTCTGGCCATCATGG*	This study, modified from [53], original sequence in italics		

**Table 5:** Strains obtained during this work tested for pathogenicity and the respective conidial density used for inoculation. Conidial density varied slightly between 1.1 × 10^6^ and 1 × 10^7^ conidia/mL. This minor difference varied depending on the capacity of each strain to sporulate. The concentrations are given for the first and second inoculation.

IPP No.	Description	Strain Designation	Conidia/mL
1562	*Phoma* sp.	LRR 12.18	5 × 10^6^–1 × 10^7^
1563	*Phoma* sp.	LRR 13.2B	1.1 × 10^6^–4.5 × 10^6^
1564	*Phoma* sp.	LRR 13.2C	1 × 10^7^
1577	*Phoma* sp.	LRR 12.20	1 × 10^7^
1575	*Phoma* sp.	LRR 12.23.1p	2 × 10^6^–4 × 10^6^
1576	*Phoma* sp.	LRR 13.2P	1 × 10^7^
1566	*Phoma* sp.	LRR 12.27	1 × 10^7^
1567	*Phoma* sp.	LRR 12.13	1 × 10^7^
1568	*Phoma* sp.	LRR 12.10	1 × 10^7^
1569	*Phoma* sp.	LRR 13.2d	1 × 10^7^
1570	*Phoma* sp.	LRR 13.48	1 × 10^7^
1571	*Phoma* sp.	LRR 13.47	1 × 10^7^
1572	*Phoma* sp.	LRR 12.28	1 × 10^7^
1573	*Phoma* sp.	LRR 13.36	1 × 10^7^
1574	*Phoma* sp.	LRR 13.37	1 × 10^7^
1585	*Phoma* sp.	LRR 12.31	1 × 10^7^

## Data Availability

The concatenated sequences of each isolate are available in fasta format in the supplementary text file.

## Supplementary Materials

The following are available online at https://www.mdpi.com/article/10.3390/pathogens10091216/s1, Table S1: Coutries and locations where maize leaf samples were taken and/or analyzed for *Phoma*-like organisms and other pathogens, supplementary file.
