# Supplementary material for: First Characterisation of the Phoma Species Complex on Maize Leaves in Central Europe
Source: Pathogens. 2021 Sep 18;10(9):1216. doi: 10.3390/pathogens10091216 (PMC8467443; doi:10.3390/pathogens10091216)
Supplement: Supplementary file 1 [file pathogens-10-01216-s001.zip › pathogens-1338171-Table S1.pdf]

Table S1. Coutries and locations where maize leaf samples were taken and/or analyzed for Phoma-like organisms and other pathogens.

Country	Locations	Locations visited*	Representative leaf samples analysed (2012-2013)
Poland	2	0	2
France	2	0	4
Austria	3	2	10
The Netherlands	6	6	23
Czech Republic	6	6	31
Germany	27	9	84
**Total**	46	23	154

* Samples were either sent by local colleagues or collected by L. Ramos-Romero by visiting the individual experimental sites
